# Dismantling the relative effectiveness of core components of cognitive behavioural therapy in preventing depression in adolescents: protocol of a cluster randomized microtrial

**DOI:** 10.1186/s12888-019-2168-6

**Published:** 2019-06-27

**Authors:** Marieke W. H. van den Heuvel, Denise H. M. Bodden, Mirjam Moerbeek, Filip Smit, Rutger C. M. E. Engels

**Affiliations:** 1 Department of Public Mental Health, Trimbos-institute (Netherlands Institute of Mental Health and Addiction), P.O. Box 725, 3500 AS Utrecht, The Netherlands; 2 Child and Adolescent Studies, Utrecht University, P.O. Box 80140, 3508 TC Utrecht, The Netherlands; 3 Behavioral Science Institute, Radboud University Nijmegen, P.O. Box 9104, 6500 HE Nijmegen, The Netherlands; 4 Methodology and Statistics, University of Utrecht, P.O. Box 80.140, 3508 TC Utrecht, The Netherlands; 5 Department of Clinical, Neuro and Developmental Psychology and Department of Epidemiology and Biostatistics, Amsterdam Public Health research institute, VU University Medical Center, PO Box 7057, 1007 MB Amsterdam, The Netherlands; 6 Erasmus School of Social and Behavioural Sciences, Erasmus University, P.O. Box 1738, 3000 DR Rotterdam, The Netherlands

**Keywords:** Prevention, Depression, Adolescents, Cognitive behavioural therapy, Comparative effectiveness research, Cost effectiveness

## Abstract

**Background:**

Both depressive disorder and subclinical depressive symptoms during adolescence are a major public health concern. Therefore, it is important that depression is detected at an early stage and is treated preventively. Prevention based on the principles of Cognitive Behavioural Therapy (CBT) has proven to be the most effective, however research has mainly focused on the effectiveness of “prevention packages” consisting of multiple CBT-components, rather than on the distinct CBT-components. This study will evaluate the relative effectiveness of four core components of CBT (cognitive restructuring (CR), behavioural activation (BA), problem solving (PS) and relaxation (RE)). In addition the relative (cost-)effectiveness of four different sequences of these components will be evaluated: (1) CR – BA – RE – PS, (2) BA – CR – RE – PS, (3) PS – GA – CR – RE and (4) RE – PS – BA – CR.

**Methods:**

We will perform a non-blinded multisite cluster randomized prevention microtrial with four parallel conditions consisting of the four sequences. The four sequences of components will be offered in groups of high school students with elevated depressive symptoms. For each CBT-component a module of three sessions is developed. Assessments will be conducted at baseline, after each CBT-component, prior to each session, at post-intervention and at 6-month follow-up. Potential moderators and mediators will be evaluated exploratively to shed light on for whom the (sequences of) CBT-components are most effective and how effects are mediated.

**Discussion:**

The potential value of the study is insight in the relative effectiveness of the four most commonly used CBT-components and four different sequences, and possible moderators and mediators in the prevention of depression among adolescents. This knowledge can be used to optimize and personalize CBT-programs.

**Trial registration:**

The study is registered in the Dutch Trial Register (Trial NL5584 / NTR6176) on October 13, 2016.

## Background

Depressive disorder during adolescence is a major public health concern, as it is one of the most prevalent mental disorders among adolescents (e.g., [1, 2]). The annual prevalence of major depressive disorder is approximately 7.4% [1]. Lifetime prevalence of depression in adolescents is estimated at 10.6% [1]. These percentages do not even include adolescents with subclinical depressive symptoms. Adolescent depression is associated with a range of adverse consequences such as social isolation [3], poor academic functioning [4], substance abuse [5], suicidal behaviours [6], comorbid psychiatric diagnoses [7, 8] and high societal costs [9]. Not only depressive disorders but also subclinical levels of depressive symptoms put adolescents at risk for poor social and academic functioning [10, 11]. Furthermore, it puts adolescents at risk for subsequent depressive disorders later in life [12]. Therefore, it is important that depression is detected at an early stage and is treated preventively. Programs based on the principles of Cognitive Behavioural Therapy (CBT) have proven to be the most effective and most applied in preventing depression among adolescents (e.g., [13]). Thus far, research has mainly focused on effectiveness of prevention programs as “packages” consisting of multiple CBT-components, rather than on the distinct CBT-components. Little is known about *which* specific components of CBT contribute to the effectiveness of CBT-programs and *in what order* these components should be offered (i.e., what their optimal sequencing is). Most common CBT-components in current prevention programs for depression in adolescents are cognitive restructuring, behavioural activation, problem solving and relaxation (e.g., [14–16]). However, few studies have examined the effectiveness of these specific CBT-components and its sequencing [17]. The planned study is aimed at dismantling the relative effectiveness of core components of CBT and its sequencing in the prevention of depression among adolescents.

Cognitive restructuring is often seen as the fundamental component of CBT. Cognitions play an important role in the theoretical explanation of mood disorders (e.g., [18]). Beck et al. [18] suggested that the way people think and process information in response to stressors, is a primary determinant of mood. In depressed individuals, negative thoughts elicit a depressive mood, while positive thoughts elicit happier moods. Negative thoughts are generated by dysfunctional, negative and often unrealistic beliefs about the self, the world and the future (the cognitive triad). The aim of cognitive restructuring is to challenge these negative beliefs and to generate more realistic thoughts which will help to improve mood [18, 19]. There is debate about the necessity of focusing on cognitions in treating depression in adolescents as their cognitive ability to reflect on their own core beliefs has not yet fully developed (e.g., [20, 21]). This might limit the application of cognitive restructuring in this age group. Yet, there is some evidence for the effectiveness of cognitive restructuring in preventing (and treating) depression among adolescents. A recent meta-analysis showed that preventative and curative CBT including challenging thoughts (which is part of cognitive restructuring) is associated with better outcomes than CBT without this component, but only on the long term [22]. Another meta-analysis showed similar effects of interventions (preventive and curative) emphasizing cognitive change (e.g. CBT and cognitive restructuring treatments) and interventions without cognitive emphasis (e.g. relaxation training) [23].

The CBT-component behavioural activation is theoretically based on behavioural models of depression (e.g., [24, 25]), wherein it is suggested that depression is caused or maintained by a lack of experiencing positive reinforcement from the environment. This may occur because people with depression lack the social skills required to elicit rewarding interactions with others and often show avoidant behaviours [25]. Behavioural activation is aimed at increasing engagement in activities that evoke positive reinforcement [26]. Behavioural activation has proven to be an effective component in preventing (and treating) adolescents’ depression (e.g., [22, 27–31]). For example, the meta-analysis of Oud et al. [22] demonstrated that preventive and curative CBT including behavioural activation (in addition to challenging thoughts, see previous paragraph) resulted in better outcomes on the long term than CBT without behavioural activation. Besides, a Randomized Controlled Trial (RCT) showed that behavioural activation (as a standalone program in only five sessions) was effective in reducing depressive symptoms compared to no treatment in a sample of late adolescents (aged 18–19) [30].

Problem solving refers to the cognitive-behavioural process whereby people attempt to identify or discover adaptive solutions to cope with specific problems arising in daily living [32]. According to D’Zurilla and Goldfried [33] problem solving comprise five different processes, i.e. problem orientation, operationalizing problems/goals, generating solutions, decision making, and implementing and evaluating solutions. Longitudinal studies have shown that deficiencies in any of these processes play a role in the etiology of depression [34, 35]. Problem solving is directed at changing someone’s overall problem-solving orientation, including the accompanying problem-solving techniques [15, 33]. In the context of prevention, problem solving has not (yet) been proven effective. A RCT showed that five weekly sessions of Problem Solving Therapy (provided as guided online self-help) were not more effective in reducing symptoms than a wait list control [36]. However, this result may be due to limitations of the study (e.g., limited power) [36]. It could be expected that problem solving is important in the context of preventing depression among adolescents as adolescence is a challenging and stressful period wherein people are faced with a lot of developmental challenges (e.g., making school/career decisions, developing new friendships, exploring self-identity, achieving autonomy). The component of problem solving focuses on improving problem solving skills to deal effectively with these challenges [37]. In the context of treatment, problem solving has proven to be effective on adolescents’ depression. Kennard et al. [14] showed that CBT including problem solving was associated with better treatment outcomes than CBT without problem solving. Chronically depressed adolescents who received CBT including problem solving were 2.3 times more likely to have a positive response than those who received CBT without problem solving [14].

The CBT-component relaxation is based on the diathesis-stress model for depression [38]. The model states that stress may activate a diathesis or vulnerability, transforming a predisposition for depression into an actual depression [38]. Relaxation is aimed at developing strategies to cope with stress and to reduce its negative effect on a someone’s vulnerability, for example through relaxation exercises [38]. The effect of relaxation techniques on depressive symptoms has rarely been examined in adolescents in both prevention and treatment settings. One study showed equivalent effects of relaxation training and CBT (both 10 sessions) in treating depression among adolescents [39].

Besides the specific components, current CBT-protocols do not have a fixed sequence of components. Even within one session often multiple components are offered (see for example Coping with Depression course for Adolescents (CWD-A), [40]). Nevertheless, patterns can be distinguished. A quick review of four Dutch CBT-protocols (The D(o) epression course (derived from CWD-A), [41]; Pak aan (derived from Taking Action), [42]; Op Volle Kracht (derived from the Penn Resiliency Program), [43]; and Head up, [44]) showed that cognitive restructuring is offered throughout all phases of treatment, while behavioural activation is addressed in the early and middle phase of treatment. The component of problem solving is offered in the middle and final phase, while relaxation techniques are taught in the middle phase. Dobson [19] describes that a typical course of CBT is composed of behavioural activation and problem solving in the early phase of treatment and cognitive restructuring in the middle and final phase. Others suggest that CBT should not consist of a fixed sequence of components, but that the order of components should be based on individual client characteristics and needs [45]. An example of such a personalized modular approach is MATCH-ADTC (Modular Approach to Therapy for Children with Anxiety, Depression, Trauma, or Conduct Problems) by Chorpita and Weisz [46], which allows great flexibility in the sequence of modules (and thus components) based on a flowchart. To the best of our knowledge, no studies investigated whether or not the sequence of CBT-components is relevant for the effect of CBT on depressive symptoms.

In sum, we know that CBT is effective in the prevention of depression among adolescents, but we do not know which components contribute most to its effectiveness and what the optimal sequencing of components is [47]. Therefore, the current study aims to explore (1) the relative effectiveness of the four most commonly used CBT-components in the prevention of depression among adolescents (namely cognitive restructuring (CR), behavioural activation (BA), problem solving (PS) and relaxation (RE)) and (2) the relative (cost-)effectiveness of four sequences of CBT-components. First, we will examine and compare the effectiveness of the four most commonly used CBT-components. We hypothesize that all components are effective on adolescents’ depressive symptoms, but that some components are more effective than others. Because of insufficient evidence, we have no specific assumptions about which components are more effective. Second, we will examine and compare the effectiveness of four sequences of the most commonly used CBT-components. As it is impossible to test all 24 possible combinations because of limited time and budget, we will only investigate the three most logical sequences (condition 1: CR – BA – RE – PS; condition 2: BA – CR – RE – PS; condition 3: PS – GA – CR – RE) and the least logical sequence (condition 4: RE – PS – BA – CR). These four sequences are based on current CBT-protocols, theory and close consultation with mental health professionals. We hypothesize that all sequences are effective on adolescents’ depressive symptoms, but that some sequences are more effective than others. We expect that the conditions 1, 2 and 3 are more effective than condition 4. Furthermore, potential moderators (e.g. severity of depressive symptoms, comorbidity and demographics) and mediators (focusing on the four components namely negative cognitive errors, behavioural activation, problem solving skills and relaxation) will be included to explore for whom and how a component or combination of components works. At last, the role of non-specific treatment variables (client expectancy of treatment, therapeutic alliance, groups cohesion, cooperation with treatment, treatment satisfaction and treatment adherence) will be taken into account.

## Methods

The study methods and results will be reported in accordance with the CONSORT 2010 statement for reporting parallel group randomized trials [48] and the extension to cluster randomized trials [49]. The medical ethics committee CMO Region Utrecht in The Netherlands approved this study (NL59152.041.16). The study is registered in the Dutch Trial Register (Trial NL5584 / NTR6176).

### Design

The study is designed as a non-blinded multisite cluster randomized prevention microtrial with four parallel conditions to evaluate the relative effectiveness of four CBT-components and four different sequences of these components in adolescents at risk of depression. In all conditions an indicated prevention program will be offered consisting of four modules based on the four most commonly used CBT-components (cognitive restructuring (CR), behavioural activation (BA), problem solving (PS) and relaxation (RE)). Each module will consist of three sessions. The sequence of the four modules will differ per condition. The four conditions are shown in Table 1.

**Table 1:** Sequence of CBT-modules per condition

	Condition 1	Condition 2	Condition 3	Condition 4
Module 1 (session 1–3)	CR	BA	PS	RE
Module 2 (session 4–6)	BA	CR	BA	PS
Module 3 (session 7–9)	RE	RE	CR	BA
Module 4 (session 10–12)	PS	PS	RE	CR

*Abbreviations. CR* Cognitive restructuring, *BA* Behavioural activation, *PS* Problem solving, *RE* Relaxation

Assessments will be conducted at baseline (*t*_*0*_), during the intervention phase after the first module (after three sessions; *t*_*1*_), during the intervention phase after the second module (after six sessions; *t*_*2*_), during intervention phase after the third module (after nine sessions; *t*_*3*_), at post-intervention (after twelve sessions; *t*_*4*_) and at 6-month follow-up (*t*_*5*_). Additionally, prior to each session, short individual measurements will be administered in order to trace interim effects.

### Participants’ eligibility

Adolescents in all grades of secondary education (from pre-vocational education up to pre-university education) are eligible to participate in the study. Inclusion criteria for the adolescents are (1) aged between 10 and 20 years old, (2) sufficient knowledge of the Dutch language and (3) an elevated level of depressive symptoms at screening, defined as a percentile score of 76 or higher based on the Children’s Depression Inventory-2 (CDI-2; [50]) norm scores (according to gender and age). Exclusion criteria are (1) the absence of adolescents’ or parental consent (for subjects aged younger than 16), (2) currently receiving treatment for mood- or anxiety related problems and (3) suicidal ideation (expressed as a score of 2 (“I want to end my life”) on CDI-2 item 8 followed by a total score of 12 or higher on the suicide items of the Questionnaire assessing Suicide and Self Injury (in Dutch: Vragenlijst over Zelfdoding en Zelfbeschadiging; VOZZ, [51])). Adolescents who are identified with suicidal ideation (at any time point within the study), will be contacted within 48 h and referred to a general practitioner which in turn can refer to mental health care.

In order to minimize the number of false positives, an eligibility check will be conducted at baseline assessment (*t*_*0*_). However, adolescents who do not report an elevated level of depressive symptoms anymore (compared to screening) will not automatically excluded from the study, because of the episodic course of depression. In consultation with the adolescent it will be determined whether or not he/she will participate.

### Recruitment, screening and randomization

Adolescents will be recruited via secondary schools in The Netherlands. All adolescents and their parents (for subjects aged younger than 16) receive written information about the screening and the study. After receiving passive consent, adolescents will be screened with the CDI-2 [50, 52] for depressive symptoms in order to select high-risk adolescents. Students with an elevated level of depressive symptoms will be approached by email and by phone to participate in the study. Written informed consent from adolescents and parents (for subjects aged younger than 16) will be obtained by the first author (MvdH) by post or email prior to the initiation of the study. Screening will continue until the planned sample size is achieved (*n* = 256, see subheading ‘sample size’).

**Fig. 1:**
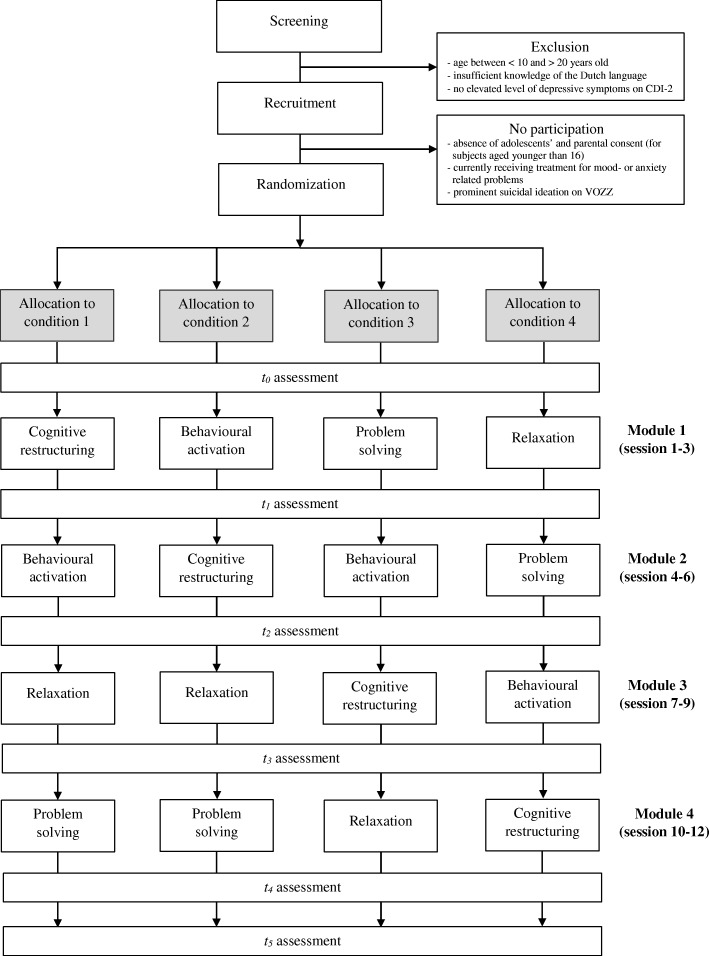
Schematic overview of the study design. Note: Each module includes three sessions

Eligible and consenting students from the same school will be stratified by gender (boy or girl) and age (12–13 years, 14–15 years, 16 years or older), because a meta-analytic review found that CBT depression prevention programs showed higher effect sizes for samples with a higher rate of female participants and samples with older participants (> 13.5 years) [53]. Then treatment groups of on average five students (from the same school) will be formed, which are the unit of randomization. The treatment groups will be randomized as a cluster by the first author (MvdH), to one of the four conditions, by computer generated block randomization (with a block size of four). Once randomized such a group of students continues as a group receiving the preventive program, with one therapist per group. Blinding of participants is not possible, like in most psychological interventions. However, allocation to the four conditions will be concealed until the introductory meeting, which takes place after the baseline assessment has been completed. The overall study design is shown in Fig. 1**.**

### Sample size

The trial is well powered with *n* = 64 in each of the four conditions (*n* = 256 in total) to detect a clinically relevant effect (mean standardized difference, *d*) of *d* ≥ 0.33 as statistically significant (at α ≤ 0.05, 2-tailed) with a power of (1-β) ≥ 0.80 when the primary outcome (depressive symptoms) is evaluated in a baseline adjusted analysis of variance (ANCOVA) while also taking into account the nesting in the data owing to the cluster randomized trial.

More specifically, the power calculation was conducted using Stata’s 14.2 sample size procedure (*sampsi*) assuming that the evaluation would be carried out in a baseline adjusted ANCOVA with one baseline assessment and one follow-up (*t*_*1*_). The correlation (*r*) between *t*_*0*_ and *t*_*1*_ (*r* = .80) was estimated from preliminary data. This indicated that 52 respondents per condition were required. In a next step, we calculated the design effect of 1.22 while taking into account the intraclass correlation coefficient (*icc*), the mean cluster size (*m*), the coefficient of variation (*cv*) of the cluster sizes. The *icc* was estimated at 0.05, *m* and *cv* where estimated from preliminary data at 5 and 0.30, respectively. Hence, 64 (52*1.22) participants per condition are needed or a total *n* of 256. We will not increase the sample size at baseline to compensate for dropout, because all analyses will carried out in agreement with the intention-to-treat principle.

### The program

The prevention program is designed for indicated prevention and will be offered in groups of about five adolescents. The program is developed by the researchers themselves, in close collaboration with CBT-therapists and experts in the field. For each of the four most commonly used CBT-components a module of three sessions is developed; one module with three sessions of cognitive restructuring (CR), one module with three sessions of behavioural activation (BA), one module with three sessions of problem solving (PS) and one module with three sessions of relaxation (RE). The modules were developed based on cognitive and behavioural theories, a taxonomy that describes the techniques that diverse CBT-components should comprise [22], current Dutch CBT-protocols (e.g., The D(o) epression course, [41]; Op Volle Kracht, [43]) and the MATCH-ADTC [46]. In each step of this developing process, a group of certified CBT-therapists and experts in the field was consulted. Also John Weisz was consulted during the process (December 2016).

The program consists of twelve sessions (4 components * 3 sessions) of 45–60 min each. Prior to the program an introductory meeting will take place which lasts 30 min. The program takes place at the participating schools directly after school, one to two times a week (depending on holidays and schools’ preferences). It will be provided by certified alumni pedagogics/psychology, who are trained in two-and a half days and supervised by psychologists. Treatment adherence of participants will be monitored by the therapists and stimulated if necessary.

### Study outcome measures

For an elaborate overview of study outcome measures, see Table 2**.**

**Table 2:** Overview of study outcome measures

Type of variable	Domain/Concept	Instrument	Items	Source			Assessment			
				A	P	T	*t*_*0*_	*t*_*1*_-*t*_*3*_	*t*_*4*_	*t*_*5*_
Primary outcome	Depressive symptoms	CDI-2 (FV) CDI-2 (SV)	28 12	x x			x	x	x	x
Secondary outcome	Depressive symptoms	NRS for core symptoms of depression ^a^	3	x			x	x	x	x
		CDI-2 parent version	17		x		x		x	x
	Depression diagnosis	K-SADS affective disorders		x	x		x		x	x
	Top three problems	TP measure^a^	3	x			x	x	x	x
	Suicidal ideation (if score 2 on item 8 CDI-2)	VOZZ suicide items	8	x			x	x	x	x
	Health-related quality of life	EQ-5D-Y	6	x	x		x		x	x
Cost-effectiveness	Healthcare costs	Cost diary	21		x		x		x	x
Moderators	Depression severity	K-SADS		x	x		x		x	x
	Comorbidity	BPM	19	x	x		x		x	x
	Demographics adolescent/parent/therapist			x	x	x	x			
Mediators	Negative cognitive errors	CNCEQ-R (FV) CNCEQ-R (SV)	16 5	x x			x	x	x	x
	Behavioural activation	BADS (FV) BADS (SV)	25 9	x x			x	x	x	x
	Problem solving skills	SPSI-R	10	x			x	x	x	x
	Relaxation	PSS-10	10	x			x	x	x	x
		NRS for relaxation	1	x			x	x	x	x
Treatment characteristics	Current and previous treatment	VEHI	6	x			x			x
	Expectancy of treatment	PETS	7	x			x			
	Therapeutic alliance	TASC-r	12	x		x		x	x	
	Groups cohesion	GCQ-s	12	x				x	x	
	Cooperation with treatment	CWT	5			x		x	x	
	Satisfaction treatment	SSS	3	x					x	

*Abbreviations*: *A* Adolescent, *FV* Full-length version, *NRS* Numerical Rating Scale, *P* Parents, *SV* Short version, *T* Therapist, *t*_*0*_ Baseline assessment, *t*_*1–3*_ Intermediate assessments, *t*_*4*_ Post-assessment, *t*_*5*_ 6-month follow-up assessment.

^a^These questionnaires will also be administered prior to each session

### Screening measures

To assess the eligibility to participate, adolescents will be screened for *depressive symptoms* using a self-report questionnaire, the CDI-2 [50] which includes 28 items. All items offer three graded options from 0 to 2, of which one is chosen (e.g., 0 = “I am sad once a while”, 1 = “I am sad many times”, 2 = “I am sad all the time”), with higher scores indicating more depressive symptoms according to the adolescent. Item 8 measures the presence of *suicidal ideation* (0 = “I don’t think about ending my life”, 1= “I think about ending my life, but I would never do”, 2 = “I want to end my life”= 2). In case a participant scores 2 on this item, the suicide items of the VOZZ [51] will be administered to assess suicidal ideation more extensively. The VOZZ consists of 39 items of which 8 items measure past and present suicidality, therefore only these items will be administered. Items are rated on a 5-point scale, ranging from “never/no day” to “very often/every day”, with higher scores indicating more self-reported suicidal ideation.

### Primary outcome measure

The degree of *depressive symptoms* in adolescents will be measured with the CDI-2 [50], as described in the previous section. Both the full-length version (28 items) and the short version (12 items) will be used. The full-length version has good internal consistency, test-retest reliability and convergent validity [50]. For the short 12-item version, Dutch psychometric properties have not been studied yet. Based on the dataset of the Dutch CDI-2 manual, we calculated the internal consistency of the short 12-version in the general population of adolescents, which was acceptable (α = .76, *n* = 2246) [50].

### Secondary outcome measures

In addition to the CDI-2, three Numerical Rating Scales will be used to measure the *core symptoms of depression* (depressed/irritable mood and loss of interest or pleasure in nearly all activities) in the last week, ranging from 0 to 10. The first scale focuses on depressed mood (0 = “very depressed”, 10 = “very happy”), the second on irritable mood (0 = “very irritated”, 10 = “very relaxed”) and the third on loss of interest or pleasure (0 = “no pleasure at all”, 10 = “a lot of pleasure”). The scales are developed by the researchers themselves, based on the core symptoms of Major Depressive Disorder specified in the Diagnostic and Statistical Manual of Mental Disorders, Fourth Edition, Text Revision (DSM-IV-TR; [54]).

The degree of *depressive symptoms of the adolescent according to the parent* will be measured with the CDI-2 parent version that contains 17 items [50]. Items are rated on a 4-point scale ranging from “not at all” to “almost always”, with higher scores indicating more depressive symptoms according to the parent. The internal consistency and convergent validity are qualified as good [8].

The presence of a *depression diagnosis* will be measured by a semi-structured diagnostic interview, the Kiddie-Schedule for Affective Disorders and Schizophrenia, present and lifetime version (K-SADS; [55, 56]). The view of the adolescent, the parent and the independent clinician will be taken into account. Only the section of affective disorders will be assessed. In previous research, the concurrent and convergent validity of the K-SADS was supported [55, 57], the interrater agreement was high (range: 93 to 100%) and test-retest reliability was excellent (.77 to 1.00) [55]. The K-SADS will be assessed by independent master students pedagogics/psychology and research assistants who will be trained and supervised in the K-SADS and depressive symptomatology. They will be blind to the allocation of the participants.

The *top three problems* of adolescents will be measured with the Top Problems (TP) measure [58]. It measures adolescents’ problems of greatest concern at baseline assessment. Adolescents are asked to list problems, they are concerned about most. When the list is complete, the interviewer will obtain severity ratings for each problem (“How big of a problem is this for you?”) on a scale ranging from 0 (not at all) to 10 (very, very much). Than the interviewer will repeat all problems that the adolescent has identified and asks which one “is the biggest problem right now?”. The problem identified will be assigned as rank 1; then the interviewer asks for the next biggest problem (rank 2), and the next (rank 3). This will result in a ranked list of the top three problems. In our study, the top three problems will be requested once (at baseline), by independent master students pedagogics/psychology and research assistants who are blind to the allocation of the participants. At subsequent assessments, only the severity of the top three problems will be rated by the adolescent. Psychometric properties of the TP measure are qualified as good [58].

As described in the previous section, *suicidal ideation* will be measured with item 8 of the CDI-2 and 8 items of the VOZZ [51]. The VOZZ has good internal consistency and test-retest reliability [51].

The Dutch version of the EQ-5D-Y [59] will be used to establish *health-related quality of life* as expressed in quality adjusted life years (QALYs), with parallel forms for adolescent and parent report. The questionnaire comprises five dimensions: Mobility, Self-care, Usual activities, Pain/discomfort and Anxiety/depression. Each dimension has three levels: no problems (1), some problems (2) and a lot of problems (3), with higher scores indicating a lower quality of life of the adolescent. The questionnaire also comprises a Visual Analogue Scale (ranging from 0 to 100) that records the adolescents’ health, where 0 is labelled as “worst imaginable health state” and 100 as “best imaginable health state”. The reliability and validity of the questionnaire have been established [60].

*Healthcare costs* will be measured by registration of health care usage and costs of the last three months in a cost diary reported by the parent, which has been used in previous research on anxiety and depression [9, 61, 62]. Health care usage and costs that will be registered are direct health care costs (e.g., psychologist, general practitioner and medication), direct non-health care costs (e.g., informal care), indirect costs (monetary value of production losses caused by absence and reduced productivity and school) and out-of-pocket costs (e.g., own contribution and transport costs). Costs will be calculated from the mental health care system’s perspective and from a societal perspective.

### Moderators

The *severity of the depression* will be rated on the basis of the number of depressive symptoms (five to six symptoms: mildly depressed, six to eight symptoms: moderately depressed, eight to nine symptoms: severely depressed), the nature of symptoms (when suicidal ideation is present, the severity will be rated as severe) and the interference with daily functioning in different contexts such as at school, at home and in social life (one life area: mildly depressed, two to three life areas: moderately depressed, four life areas: severely depressed) as suggested by the Dutch guideline for depression in youth [63]. The rating will be done by independent master students pedagogics/psychology and research assistants, who are blind to the allocation of the participants, based on the results of the K-SADS interview [55, 56].

*Comorbidity* will be assessed with the Brief Problem Monitor [1], with parallel forms for adolescent and parent report. The questionnaire contains 19 items and comprises three scales: Internalizing, Externalizing and Attention Problems. Items are rated on a 3-point scale ranging from “not true” to “very true”, with higher scores indicating more comorbid problems. Psychometric properties are qualified as good [64, 65].

*Demographic information* of the adolescent, parents and therapist will be gathered by adding questions to the self-report questionnaires about gender, age, ethnicity and education level.

### Mediators

*Negative cognitive errors* will be measured with the Children’s Negative Cognitive Errors Questionnaire-Revised (CNCEQ-R; [66]), to track changes in the assumed mediator of cognitive restructuring, namely negative cognitive errors. The questionnaire comprises 16 items, which are divided into five categories: Underestimation of the ability to cope, Personalizing without mind reading, Mind reading, Selective abstraction and Overgeneralizing. Items are rated on a 5-point scale ranging from “not at all like I would think” to “almost exactly like I would think”, with higher scores indicating more self-reported negative cognitive errors. For the total score, high test-retest reliability and good internal consistency have been established [66]. Because of limited time at the intermediate assessments (*t*_*1*_ - *t*_*3*_), we will only administer five items of the questionnaire (item 1, 4, 5, 9 and 14). For each subscale we selected the item with the highest loading based on the factor analysis of Maric et al. [66].

*Behavioural activation* will be assessed with the Behavioral Activation for Depression Scale (BADS; [67]), to measure changes in activation and avoidance. The self-report questionnaire contains four scales: Activation, Avoidance/rumination, Work/school impairment and Social impairment. Both the full-length (25 items) and the short version (9 items) will be used. The short version only contains the scales Activation and Avoidance. Items are rated on a 7-point scale ranging from “not at all” to “completely”. For all subscales, higher scores mean greater intensity on a particular dimension (e.g., the higher the score on the Activation subscale, the more activation). Psychometric properties for both the full-length and the short version are qualified as acceptable to good [68, 69].

*Problem solving skills* will be assessed with the 10-item version of the Social Problem Solving Inventory-Revised (SPSI-R; [70]), to track changes in someone’s approach toward problem solving in daily life. Items relate to five subscales: Positive problem orientation, Negative problem orientation, Rational problem solving, Impulsivity/carelessness style and Avoidance style. Items on this self-report questionnaire are rated on a 5-point scale ranging from “not at all true” to “extremely true of me”. For all subscales, higher scores reflect greater intensity on a particular dimension (e.g., the higher the score on the Positive problem orientation subscale, the more positive problem orientation someone reports). Psychometric properties have demonstrated good reliability and validity [71].

*Relaxation* will be measured with the Perceived Stress Scale (PSS-10; [72]), to measure changes in the degree to which someone appraises situations as stressful. The scale consists of 10 items, which are rated on a 5-point scale ranging from “never” to “very often”, with higher scores indicating more self-reported stress. Psychometric properties are qualified as acceptable [73]. In addition to the PSS-10, a Numerical Rating Scale from 0 to 10 will be used to measure relaxation in the last week, developed by the researchers themselves (0 = “very stressed”, 10 = “very relaxed”).

### Treatment characteristics

*Current and previous treatments* for psychological problems, including complementary and self-help treatments, will be administered with the inventory of History of Treatments (VEHI; [74]). This questionnaire consists of 6 items.

*Treatment integrity* will be established by rating two randomly chosen sessions that are video or audiotaped. Ratings will be done by two independent researchers and interrater reliability will be established.

In the context of this study, other treatment characteristics will not be further described.

### Incentives

To motivate participation in the assessments, we offer adolescents and parents incentives of respectively 35 and 10 euros in total, regardless of study condition. Adolescents will receive 5 euros per assessment and 5 euros bonus after completing all six assessments. Parents will receive 10 euros after completing all three assessments.

### Data analysis

All analyses will be conducted according to the intention-to-treat principle (i.e., all participants, as randomised). Clustering of data will be taken into account, considering that small treatment groups of students (from the same school) will be randomized as a cluster to one of the four conditions. For all analyses multilevel mixed modelling in Stata [75] and Mplus [76] will be used. The four conditions will be included as fixed effects (i.e., by using dummy variables). For treatments groups random effects will be included [77]. Finally, the schools will be included by using fixed rather than random effects, because the between-school variability cannot be estimated well with the small number of available schools (we have included 11 schools so far). Possible baseline differences between the four conditions in depressive symptoms and demographic variables will be analysed. Variables that show differences between the four conditions will be entered as covariates in all models. When the data collection is complete, all members of the research team (MvdH, DB, FS, RE) will have access to the full dataset.

### Main effect of each of the CBT-components

To examine and compare the effectiveness of each of the distinct CBT-components (cognitive restructuring, behavioural activation, problem solving and relaxation), depressive symptoms at intermediate assessment 1 (*t*_*1*_) will be used as dependent variable to measure the effect directly after receiving the first module.

### Main effect of different sequencing

To investigate and compare the effectiveness of the four different sequences of CBT-components, depressive symptoms at post-assessment (*t*_*4*_) and 6-month follow-up (*t*_*5*_) will be used as dependent variables to measure the effect directly after treatment and six months after treatment. Marginal means will be computed under the linear mixed model and these will be used to graph the impact on depressive symptom levels when the CBT-components are being offered sequentially over time.

### Moderation

To explore effect moderation, baseline depression severity, comorbidity and the adolescents’ demographics will be used.

### Mediation

Mediation analyses will be performed to investigate whether negative cognitive errors, behavioral activation, problem solving skills and relaxation are mediators in the association between the type of CBT-component and depressive symptoms (at *t*_*1*_) and between the sequence of CBT-components and depressive symptoms (at *t*_*4*_ and *t*_*5*_).

### Cost-effectiveness of different sequencing

Cost-effectiveness analyses will be conduct in accordance with the CHEERS statement [78]. Cost data will be interpolated to a period of nine months under the assumption that data obtained with the cost diaries are representative for the periods in between. Binary logistic regression will be used to assess differences between the conditions in the percentage of adolescents below the percentile score of 76 based on the CDI-2 [50] norm scores (according to gender and age) and depression-free adolescents based on the K-SADS interview [55, 56]. In order to get insight in the uncertainty surrounding subtotal and total costs and due to highly skewed cost distributions, bootstrap simulations will be conducted. The bootstrap method estimates the sampling distribution of a statistic through a large number of simulations, based on re-sampling with replacement [79]. The results based on 1000 bootstrap replications of the costs of the four conditions will be used to calculate 95% confidence intervals (95% CI) around the cost-differences, based on the 2.5th and 97.5th percentiles. Bootstrap simulations will also conducted in order to quantify the uncertainty around the incremental cost-effectiveness ratio (ICER) [80], yielding information about the joint distribution of cost and effect differences. The bootstrapped cost-effectiveness ratios will be subsequently plotted in a cost-effectiveness plane, in which the vertical line reflects the difference in costs and the horizontal line reflects the difference in effectiveness. The choice of treatment depends on the maximum amount of money that society is prepared to pay for a gain in effectiveness, which is called the ceiling ratio. Therefore, the bootstrapped ICERs will be depicted in a cost-effectiveness acceptability curve showing the probability that a condition is cost-effective using a range of ceiling ratios. Also secondary (including QALYs based on the EQ-5D-Y) and sensitivity analyses will be performed to test the robustness of the results.

## Discussion

This study protocol presents the design of a multisite cluster randomized prevention microtrial, which will evaluate the relative effectiveness of four of the most commonly used CBT-components in the prevention of depression among adolescents and different sequences of these components. In addition, the cost-effectiveness of the different sequences will be examined. Furthermore, potential moderators and mediators will be included to explore for whom and how a given component or set of components is effective. Also the role of non-specific treatment variables will be taken into account.

### Strengths and limitations

To the best of our knowledge, this is the first experimental study that dismantles the effectiveness of the four most commonly used CBT-components in the prevention of depression among adolescents (i.e., cognitive restructuring, behavioural activation, problem solving and relaxation) in order to identify the most effective components of CBT and the optimal sequence of these components. An additional strength of the study is that we will not only focus on the effectiveness of the various components and sequences, but that also potential moderators and mediators will be explored. This will shed light on for whom the different components and sequences are most effective and how effects are mediated. Besides, the cost-effectiveness of the different sequences of components will be investigated, which is important because of diminishing budgets in mental health care. Furthermore, not only self-report, but also a clinical interview (K-SADS) will be conducted by independent and blinded assessors which enables us to examine the presence of a depressive diagnosis. In addition, the TP measure will be used which can complement standardized assessment as it comprises a client-guided approach [58].

The study also has limitations. We will not include a control group, limiting the extent to which a decrease in depressive symptoms in the four intervention groups can be uniquely ascribed to the prevention program. The reason for this is that several meta-analyses and reviews have already established the superior effect of CBT in comparison to waitlist and placebo (e.g., [81–83]). Another limitation is the design of a cluster randomized trial, because of the great risk on baseline imbalance between study arms [84]. We will try to reduce this risk by stratifying students by gender and age. The study’s power is also a limitation. Although the study is well powered on the main effects, the power might be too low for conducting moderation analyses. Therefore, these analyses will be done exploratory. At last, given previous prevention trials, we expect a high number of dropout in both the assessment and intervention phase, which will have consequences for the effectiveness [85]. In order to motivate adolescents to participate in the assessments, we will provide incentives for participation. Besides, all treatment drop-outs will be asked to complete the post- and 6-month follow-up assessments. To prevent the effect of dropout on the effectiveness, all analyses will be conducted according to the intention-to-treat principle.

### Implications for practice

The potential value of the study is that we gain insight in the relative effectiveness of the four most commonly used CBT-components and different sequences of components, and possible moderators and mediators in the prevention of depression among adolescents. This knowledge can be used to develop recommendations to optimize and personalize CBT-programs in the prevention of depression in adolescents. Knowing what works for whom and how in preventing depression among youth with CBT, could enable a matching process between the individual client and the most beneficial components. For example, some components could be omitted to prevent depression in some clients while maintaining the effectiveness and increasing the cost-effectiveness. In such a way, we can personalize indicated prevention for depression in youth that meets the individual needs and thereby enhance effectiveness.

## Data Availability

Not applicable.

## Abbreviations

BA: Behavioural activation
BADS: Behavioral Activation for Depression Scale
BPM: Brief Problem Monitor
CBT: Cognitive Behavioural Therapy
CDI-2: Children’s Depression Inventory-2
CMO: Commissie Mensgebonden Onderzoek (in English: Committee on Research Involving Human Subjects)
CNCEQ-R: Children’s Negative Cognitive Errors Questionnaire-Revised
CR: Cognitive restructuring
CWD-A: Coping with Depression course for Adolescents
CWT: Cooperation With Treatment
DSM-IV-TR: Diagnostic and Statistical Manual of Mental Disorders, Fourth Edition, Text Revision
EQ-5D-Y: EuroQol, Five Dimensions, Youth
GCQ-s: Group Climate Questionnaire-short
ICER: Incremental cost-effectiveness ratio
K-SADS: Kiddie-Schedule for Affective Disorders and Schizophrenia, present and lifetime version
MATCH-ADTC: Modular Approach to Therapy for Children with Anxiety, Depression, Trauma, or Conduct Problems
NRS: Numerical Rating Scale
NTR: Nederlands Trial Register (in English: Dutch Trial Register)
PETS: Parent Expectancies for Therapy Scale
PS: Problem solving
PSS-10: Perceived Stress Scale
QALYs: Quality adjusted life years
RCT: Randomized Controlled Trial
RE: Relaxation
SPSI-R: Social Problem Solving Inventory-Revised
SSS: Service Satisfaction Scale
TASC-r: Therapy Alliance Scale for Children-revised
TP: Top Problems
VEHI: Vragenlijst Eerdere Hulp en Interventies (in English: Inventory of History of Treatment)
VOZZ: Vragenlijst over Zelfdoding en Zelfbeschadiging (in English: Questionnaire assessing Suicide and Self Injury)
